# A Systematic Review and Meta-Analysis on the Efficacy of Puerarin Injection as Adjunctive Therapy for Unstable Angina Pectoris

**DOI:** 10.3389/fcvm.2022.763567

**Published:** 2022-02-24

**Authors:** Huikai Shao, Yang Huang, Dongsheng Xu, Shengfeng Huang, Rongsheng Tong

**Affiliations:** ^1^Department of Pharmacy, Sichuan Academy of Medical Sciences & Sichuan Provincial People's Hospital, Chengdu, China; ^2^Personalized Drug Therapy Key Laboratory of Sichuan Province, School of Medicine, University of Electronic Science and Technology of China, Chengdu, China; ^3^Key Laboratory of Molecular Target & Clinical Pharmacology and the State & NMPA Key Laboratory, School of Pharmaceutical Sciences & the Fifth Affiliated Hospital, Guangzhou Medical University, Guangzhou, China; ^4^Institute of Pharmaceutical Analysis, College of Pharmacy, Jinan University, Guangzhou, China

**Keywords:** puerarin injection, adjunctive therapy, unstable angina pectoris, systematic review, meta-analysis

## Abstract

**Background:**

As adjunctive therapy, puerarin injection has been widely applied for the treatment of unstable angina pectoris (UAP) in China during the past decades. However, the efficacy of puerarin injection as adjunctive therapy for UAP has not been well confirmed. The purpose of this meta-analysis was to summarize the available evidence to estimate the efficacy of puerarin injection in treating UAP.

**Objective:**

A systematic review and meta-analysis with subgroup analysis and sensitivity analysis according to the Preferred Reporting Items for Systematic Reviews and Meta-Analyses (PRISMA) principle were carried out to evaluate the efficacy of puerarin injection as adjunctive therapy in treating UAP.

**Methods:**

To obtain the published randomized controlled trials (RCTs) on puerarin injection, databases, namely, China National Knowledge Infrastructure (CNKI), Wanfang Database, Chinese Biomedical Literature Database, Sino-Med, PubMed, China Science and Technology Journal Database (VIP), Medline, Google Scholar, Cochrane Library, Chinese Science Citation Database, and Embase were systematically searched until June 2021. In this meta-analysis, Review Manager version 5.3 software and Stata version 12.0 software were employed to analyze the collected data. Based on the methodological quality, years of publications, sample size and dosages, sensitivity analysis, and subgroup analysis were performed. The GRADE assessment was conducted by the software GRADEpro version 3.6 software.

**Results:**

A total of 17 RCTs involving 1,459 patients were included in this meta-analysis. Results indicated that puerarin injection as adjunctive therapy was more superior than conventional Western medicine alone in reducing angina symptoms [risk ratio (RR) = 1.22, 95% CI 1.16 to 1.28, *Z* = 8.11, *p* < 0.00001] and improving ECG (RR = 1.32, 95% CI 1.20 to 1.44, *Z* = 6.00, *p* < 0.00001), meanwhile reducing the frequency of angina attack [mean difference (MD) = −2.22, 95% CI −2.53 to −1.90, *Z* = 13.97, *p* < 0.00001] and the duration of angina attack (MD = −2.00, 95% CI −2.39 to −1.61, *Z* = 9.99, *p* < 0.00001) for the treatment of UAP. Results from the GRADE assessment suggested that the comprehensive quality of this evidence was low.

**Conclusion:**

This meta-analysis indicated that puerarin injection was more effective than using conventional Western medicine alone in the treatment of UAP. However, because of the low methodological quality of the included RCTs, more evidence was still needed to verify the efficacy of puerarin injection.

## Introduction

As a symptom of coronary artery disease (CAD), unstable angina pectoris (UAP) is characterized by clinical symptoms of left anterior chest pain or discomfort of adjacent areas. UAP is triggered by increased myocardial oxygen demand or decreased myocardial oxygen supply (1, 2). UAP can increase the risk of severe cardiac arrhythmias and acute myocardial infarction, which leads to sudden death (3). Owing to the complexity of UAP, lifelong medication is usually necessary for the patients. Therefore, appropriate treatment should be given to the patients timely after being diagnosed with UAP. Although conventional Western medicines, namely, β-blockers, calcium antagonists, and nitrates have been successfully used in decreasing anginal attacks, drug resistances, and adverse reactions were accompanied with these Western medicines (4, 5). Therefore, exploring a novel treatment method is of great importance for treating UAP. As adjunctive therapy, traditional Chinese medicine (TCM) has attracted great interest due to its remarkable effectiveness, high bioavailability, and swift action (6). TCM as adjunctive therapy could be a promising candidate to offer better therapeutic effects and avoid the adverse reactions caused by conventional Western medicines (6).

Puerarin (Supplementary Figure 1) injection, a natural isoflavone, was isolated from *Pueraria lobata* root (7). The concentration of commercial puerarin injection was determined as 50 mg/ml and it was usually given to the patients by intravenous drip (diluted with 250 ml of 5% glucose injection, once per day). A large number of studies have demonstrated that puerarin injection was beneficial for the cardiovascular system by expanding the coronary artery to relieve vasospasm and increasing coronary blood flow, thus improving the blood supply to ischemic myocardium (8). Puerarin injection also can be used to decrease blood pressure, heart rate, and myocardial oxygen consumption (9). Moreover, this injection plays important role in inhibiting platelet aggregation, decreasing blood viscosity, and improving microcirculation (10). To provide great efficacy, puerarin injection was usually integrated with Western medicines (nitroglycerin, isosorbide nitrate, nifedipine, aspirin, trimetazidine, metoprolol, etc.) for the treatment of UAP in China.

To date, lots of randomized controlled trials (RCTs) reported positive results on the therapeutic effect of puerarin injection for UAP. However, the efficacy of puerarin injection as adjunctive therapy for UAP has not been well evaluated. Hence, the study aimed to conduct a comprehensive and the Preferred Reporting Items for Systematic Reviews and Meta-Analyses (PRISMA)-compliant systematic review with sensitivity and subgroup analysis to validate the efficacy of puerarin injection as adjunctive therapy in treating UAP (2, 11–14).

## Methods

### Eligibility Criteria

Two authors (Shao HK and Huang Y) independently screened the eligible RCTs based on the inclusion criteria as follows: (1) RCTs investigating the therapeutic effect and safety on puerarin injection as adjunctive therapy (puerarin injection combined with Western medicines vs. Western medicines alone) in the treatment of UAP were included in this meta-analysis; (2) The diagnosis of UAP was based on “the 2002 American College of Cardiology/American Heart Association (ACC/AHA) guideline for the diagnosis and management of patients with unstable ischemic heart disease (2002 ACC/AHA)” (15) or “the WHO guideline” (1) or “the diagnostic criteria of UAP developed by the Chinese Society of Cardiology” (16). (3) Duration of the treatment was more than 10 days; (4) the numbers of patients with UAP were more than 40; and (5) symptomatic improvement, ECG improvement, frequency of angina attack (time/week), or duration of angina attacks were defined as the outcome measures.

Randomized controlled trials should be excluded, if they did not meet with the above criteria: (a) Formulation and dosages of intervention in the control and treatment groups were not provided in detail; (b) Incomplete data or duplication existed; (c) Another TCM was used; and (d) Animal or tissue cell was involved.

### Information Sources

To obtain the RCTs about the adjunctive therapy of puerarin injection for UAP, databases involving China National Knowledge Infrastructure (CNKI), Chinese Biomedical Literature Database, Google Scholar, Wanfang Database, Sino-Med, China Science and Technology Journal Database (VIP), PubMed, Medline, Cochrane Library, Embase, and Chinese Science Citation Database were systematically searched and screened. The latest search was carried out on June 28, 2021.

### Search Strategies

In English databases, the following keywords were searched in separate or joint methods: puerarin, puerarin injection, unstable angina pectoris, and angina pectoris. In Chinese databases, the following keywords were searched in separate or joint methods: Gegensu (puerarin), Gegensu Zhusheye (puerarin injection), Buwending Xinjiaotong (unstable angina pectoris), and Xinjiaotong (angina pectoris). Besides, the references in the included RCTs were also screened to obtain the eligible RCTs.

### Study Selection

Two authors (Shao HK and Huang Y) independently searched the databases to obtain the eligible RCTs according to the inclusion and exclusion criteria. Disagreements between these two authors during the process of study selection were resolved by discussion.

### Data Collection Process

One author (Shao HK) read the full text of the eligible RCTs and collected data. Another author (Huang Y) checked the accuracy and completeness of the collected data. Disagreement during this process was resolved through discussion with a third author (Xu DS). Review Manager version 5.3 software (Cochrane Collaboration, Nordic Cochrane Centre, Copenhagen, Denmark) and Stata version 12.0 software (Stata Corp LLC, College Station, Texas, USA) were adopted to analyze the collected data.

### Data Items

In the included RCTs, the following items were collected: (1) First author; (2) Publication date; (2) Sample size of patients; (3) Interventions (including the dosages and duration) in the control group and the treatment control group; (4) Outcome measures; and (5) Adverse reactions.

### Quality Assessment

Two authors (Shao HK and Huang Y) independently evaluated the methodological quality of the included RCTs with the Jadad scale (Supplementary Table 1) and its refined revision M scale (2, 12, 13, 17). In the Jadad score, the methodological quality of the included RCTs was evaluated based on the items of randomization, blinding, and withdrawals (dropouts). The range of the Jadad score was from 0 (poorest) to 5 (highest). Scores were given to the RCTs if: randomization was described, 1 score; appropriate randomization, 1 score; double-blinding was described, 1 score; appropriate double-blinding, 1 score; withdrawals or dropouts were described, 1 score. RCTs obtained 3 to 5 scores were regarded as high quality, while RCTs obtained 1 or 2 scores were regarded as low quality. Two authors (Shao HK and Huang Y) independently evaluated the methodological quality of the included RCTs using the Jadad scale and M scale (RCTs with M score above 3 were regarded as high quality). Any disagreement during this process between these two authors was resolved through discussion with a third author (Xu DS).

### Sensitivity and Subgroup Analysis

Sensitivity analysis was conducted to evaluate whether the overall therapeutic effect of puerarin injection as adjunctive therapy for UAP was impacted by the low-quality RCTs. Subgroup analysis was conducted to evaluate whether the overall therapeutic effects were homogeneous in subgroups according to sample size, publication date, and dosages of puerarin injection.

### Risk of Bias Across Studies

The funnel plot created by the software STATA version 12.0 was employed to evaluate the potential publication bias. Begg's test and Egger's test were also used to detect the potential publication bias in this meta-analysis.

### Statistical Analysis

RevMan version 5.3 was used for the statistical analysis. The dichotomous variable was represented as the pooled risk ratios (RRs) with 95%CI. The continuous variable was represented as the weighted mean difference (MD) with 95%CI. *I*^2^ statistic and the chi-squared test were employed to evaluate the heterogeneity between the included RCTs. If *I*^2^ > 50% or *p* < 0.05, it suggested that a significant statistical heterogeneity was observed and the random-effect model should be employed to evaluate the outcome measures. Otherwise, the fixed-effect model was adopted. To evaluate whether the overall effects of puerarin injection as adjunctive therapy were superior to Western medicine alone for UAP, Z-test was employed. If *p* < 0.05, it suggested that there was a significant statistical difference in this meta-analysis.

### Evidence Quality Assessment

The evidence quality assessment was independently conducted by two authors (Shao HK and Huang Y) based on the GRADE criteria (18). The evidence quality of the included outcomes was defined as high, moderate, low, and very low. The evidence of RCTs was initially considered as high-quality. The quality of each outcome was downgraded according to the following five factors: risk of bias, inconsistency, indirectness, imprecision, and publication bias. In this study, GRADEpro version 3.6.1 software was adopted for the data analysis and synthesis.

## Results

### Study Selection

**Figure 1:**
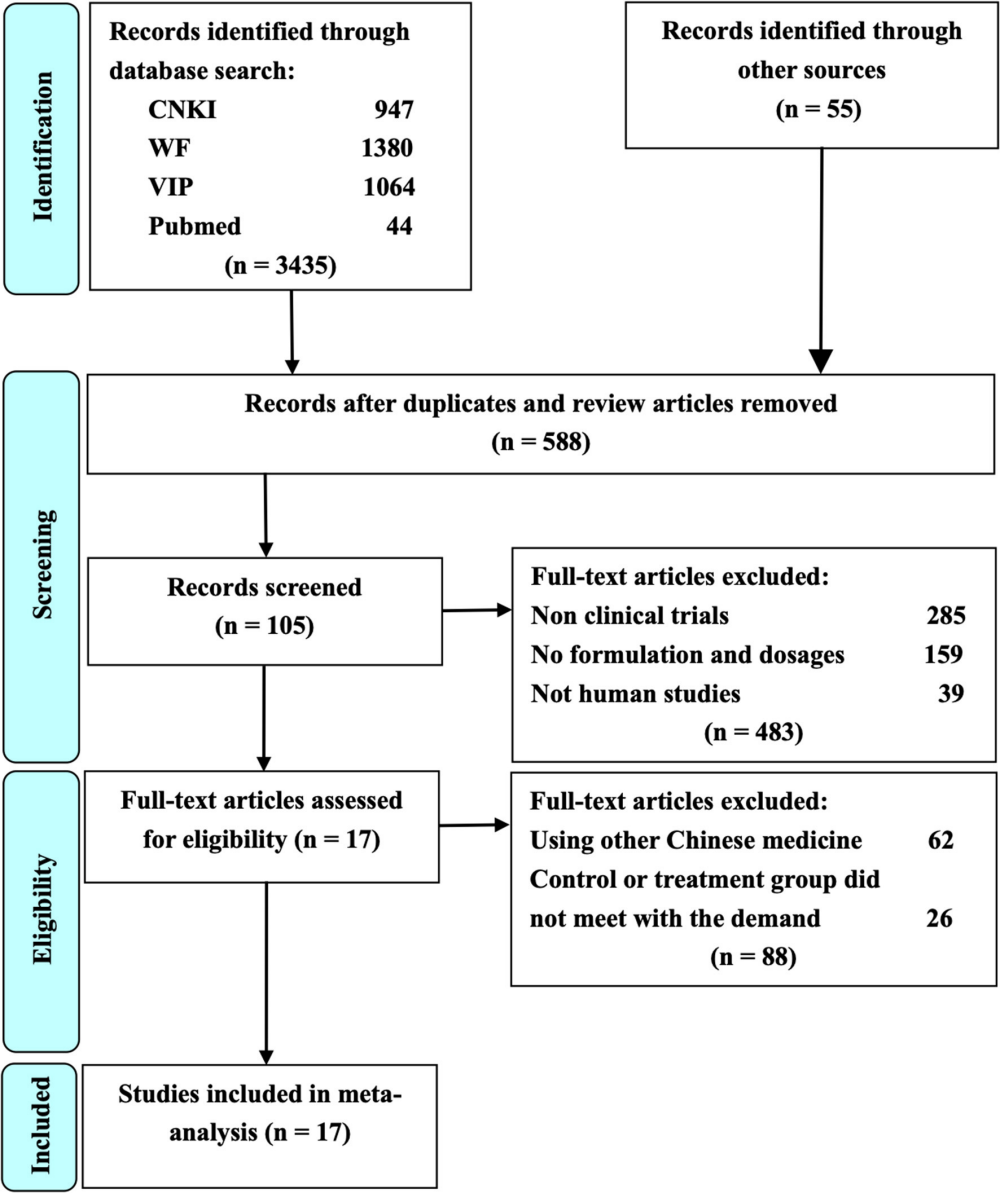
The study selection process for the identification of eligible studies. CNKI is China National Knowledge Infrastructure; WF is Wangfang Data; VIP is Chinese Scientific Journal Database; Other sources are Medline, Chinese Biomedical Literature Database, Cochrane Library, Embase, Google Scholar, Sino-Med, and Chinese Science Citation Database.

The process of study selection for the eligible RCTs is shown in Figure 1. A total of 3,490 potential studies were initially identified from the databases according to the search strategies. A total of 2902 studies were excluded because they were reviews or duplication. Full texts of 105 studies were retrieved for manually screening according to the eligibility criteria. Finally, 17 RCTs were included for further quality evaluation and meta-analysis.

### Study Characteristics

The detailed characteristics of the included 17 RCTs are given in Table 1. All the 17 RCTs were reported in Chinese language journals and the publication date was from 2000 to 2017 (19–35). The number of patients with UAP in the included RCTs ranged from 40 to 168 and the mean sample size was 85.8. The duration of the treatment was ranging from 10 to 14 days. Western medicines used in the control group included isosorbide dinitrate, nifedipine, aspirin, nitroglycerin, caltrate with vitamin D tablet, alendronate sodium tablet, metoprolol, atorvastatin calcium tablet, trimetazidine tablet, diltiazem hydrochloride tablets, coenzyme, clopidogrel, fluvastatin, isosorbide mononitrate, and low-molecular-weight heparin calcium. Combined with these Western medicines, the dosage of puerarin injection in the treatment group ranged from 60 to 500 mg per day. The symptomatic change was selected as the outcome measure in 17 RCTs. ECG was reported as the outcome measure in eight RCTs.

**Table 1:** Characteristics of RCTs on puerarin injection for unstable anginal pectoris.

**References**	**Intervention**		**Sample size**		**Course of treatment (day)**		**Outcome measures**
	**Treatment group**	**Control group**	**Treatment group**	**Control group**	**Treatment group**	**Control group**	
Cai and Liu (19)	Puerarin injection 60 mg, isosorbide nitrate 10 mg, nifedipine 15–30 mg, aspirin 50 mg, nitroglycerin 0.3–0.6 mg	Isosorbide dinitrate 10 mg, nifedipine 15–30 mg, aspirin 50 mg, nitroglycerin 0.3–0.6 mg	40	38	20	20	Symptoms
Chen and Yan (20)	Puerarin injection 200 mg, caltrate with vitamin D tablet 600 mg, alendronate sodium tablet 70 mg, nitroglycerin 1.5 mg, Metoprolol 150 mg, atorvastatin calcium tablet 20 mg, trimetazidine tablet 60 mg	Caltrate with vitamin D tablet 600 mg, alendronate sodium tablet 70 mg, nitroglycerin 1.5 mg, metoprolol 150 mg, atorvastatin calcium tablet 20 mg, trimetazidine tablet 60 mg	54	53	14	14	Symptoms
Fan and Lv (21)	Puerarin injection 500 mg, aspirin 100 mg, isosorbide dinitrate 15 mg	Aspirin 100 mg, isosorbide dinitrate 15 mg	39	40	15	15	Symptoms ECG
Guo et al. (22)	Puerarin injection 400 mg, nitroglycerin injection 5 mg	Nitroglycerin injection 5 mg	36	56	14	14	Symptoms
Huang et al. (23)	Puerarin injection 500 mg, aspirin 100 mg, isosorbide dinitrate 30 mg, nitroglycerin 10 mg	Aspirin 100 mg, isosorbide dinitrate 30 mg, nitroglycerin 10 mg	30	30	14	14	Symptoms ECG
Liang and Liu (24)	Puerarin injection 400 mg, aspirin 100–300 mg	Aspirin 100–300 mg	34	34	15	15	Symptoms ECG
Li and Ji (25)	Puerarin injection 400 mg, isosorbide dinitrate 30 mg, metoprolol 50–100 mg, aspirin 100 mg	Isosorbide dinitrate 30 mg, metoprolol 50–100 mg, aspirin 100 mg	58	44	15	15	Symptoms ECG
Liu (26)	Puerarin injection 500 mg, isosorbide dinitrate 30 mg, diltiazem hydrochloride tablets 90 mg, metoprolol 25 mg, aspirin 100 mg	Isosorbide dinitrate 30 mg, diltiazem hydrochloride tablets 90 mg, metoprolol 25 mg, aspirin 100 mg	34	34	10	10	Symptoms ECG
Meng (27)	Puerarin injection 400 mg, isosorbide dinitrate 30 mg, aspirin 100 mg, coenzyme Q10 60 mg, nitroglycerin 10 mg	Isosorbide dinitrate 30 mg, aspirin 100 mg, coenzyme Q10 60 mg, nitroglycerin 10 mg	30	30	14	14	Symptoms ECG
Wang and Jiang (28)	Puerarin injection 400 mg, isosorbide dinitrate 10 mg, aspirin 50–100 mg, nifedipine 10 mg, nitroglycerin 0.6 mg	Isosorbide dinitrate 10 mg, aspirin 50–100 mg, nifedipine 10 mg, nitroglycerin 0.6 mg	29	27	10	10	Symptoms
Xia et al. (29)	Puerarin injection 500 mg, clopidogrel 75 mg	Clopidogrel 75 mg	57	57	28	28	Symptoms
Yang and Qu (30)	Puerarin injection 500 mg, fluvastatin 40 mg, nitroglycerin 0.5 mg, aspirin 100 mg, metoprolol 50 mg	Fluvastatin 40 mg, nitroglycerin 0.5 mg, aspirin 100 mg, metoprolol 50 mg	84	84	84	84	Symptoms
Yao and Wang (31)	Puerarin injection 500 mg, aspirin 50–100 mg	Aspirin 50–100 mg	45	30	12	12	Symptoms ECG
Yuan and Ji (32)	Puerarin injection 500 mg, isosorbide dinitrate 30 mg, aspirin 50 mg, nitroglycerin 0.3–0.6 mg	Isosorbide dinitrate 30 mg, aspirin 50 mg, nitroglycerin 0.3–0.6 mg	20	20	10	10	Symptoms
Zhang (33)	Puerarin injection 500 mg, aspirin 100 mg, atorvastatin 20 mg, metoprolol 50 mg, isosorbide mononitrate 20 mg	Aspirin 100 mg, atorvastatin 20 mg, metoprolol 50 mg, isosorbide mononitrate 20 mg	80	68	14	14	Symptoms ECG
Zhang and Lao (34)	Puerarin injection 500 mg, aspirin 150 mg, fluvastatin 40 mg, low molecular weight heparin calcium 10 kU	Aspirin 150 mg, fluvastatin 40 mg, low molecular weight heparin calcium 10 kU	33	31	14	14	Symptoms
Zhang et al. (35)	Puerarin injection 500 mg, isosorbide dinitrate 30 mg, aspirin 300 mg, low-molecular-weight heparin calcium 6,150 iu, metoprolol 25 mg, nitroglycerin 0.5 mg	Isosorbide dinitrate 30 mg, aspirin 300 mg, low-molecular-weight heparin calcium 6,150 iu, metoprolol 25 mg, nitroglycerin 0.5 mg	40	40	28	28	Symptoms

### Risk of Bias in Individual Studies

To evaluate the quality of the included 17 RCTs, the Jadad scale (ranged from 0 to 5 scores) and M scale (ranged from −1 to 7 scores) were employed. As shown in Table 2, according to the Jadad scale, 3 RCTs were given 3 scores (high quality) and the rest of 14 RCTs were given 2 scores (low quality). According to the M scale, 10 RCTs were given 3 scores (low quality) and 7 RCTs were given 4 scores (high quality).

**Table 2:** Quality of the included RCTs.

**Study**	**M1**	**M2**	**M3**	**M4**	**M5**	**M score**	**J1**	**J2**	**J3**	**J Score**
Cai and Liu (19)	1	1	0	1	1	4	1	0	1	2
Chen and Yan (20)	1	1	0	1	1	4	1	0	1	2
Fan and Lv (21)	1	1	0	1	1	4	1	0	1	2
Guo et al. (22)	1	1	0	1	1	4	1	0	1	2
Huang et al. (23)	1	1	0	1	1	4	1	0	1	2
Liang and Liu (24)	1	1	0	1	0	3	1	0	1	2
Li and Ji (25)	1	1	0	1	0	3	1	0	1	2
Liu (26)	1	1	0	1	1	4	1	0	1	2
Meng (27)	1	1	0	1	0	3	1	0	1	2
Wang and Jiang (28)	1	1	0	1	0	3	1	0	1	2
Xia et al. (29)	1	1	0	1	0	3	2	0	1	3
Yang and Qu (30)	1	1	0	1	0	3	2	0	1	3
Yao and Wang (31)	1	1	0	1	1	4	1	0	1	2
Yuan and Ji (32)	1	1	0	1	0	3	1	0	1	2
Zhang (33)	1	1	0	1	0	3	1	0	1	2
Zhang (33)	1	1	0	1	0	3	1	0	1	2
Zhang et al. (35)	1	1	0	1	0	3	2	0	1	3

### Angina Symptoms

**Figure 2:**
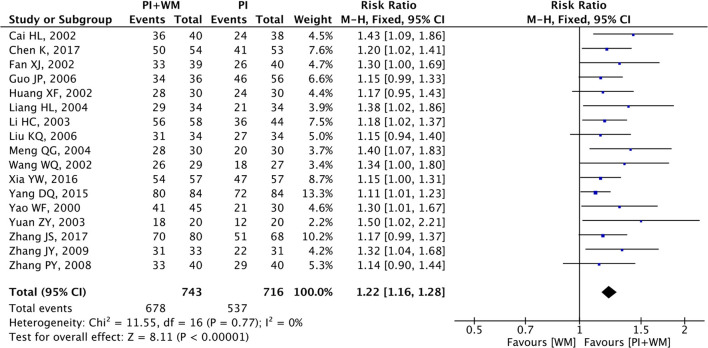
Forest plot of outcome measure symptoms. PI is puerarin injection and WM is Western medicine.

**Figure 3:**
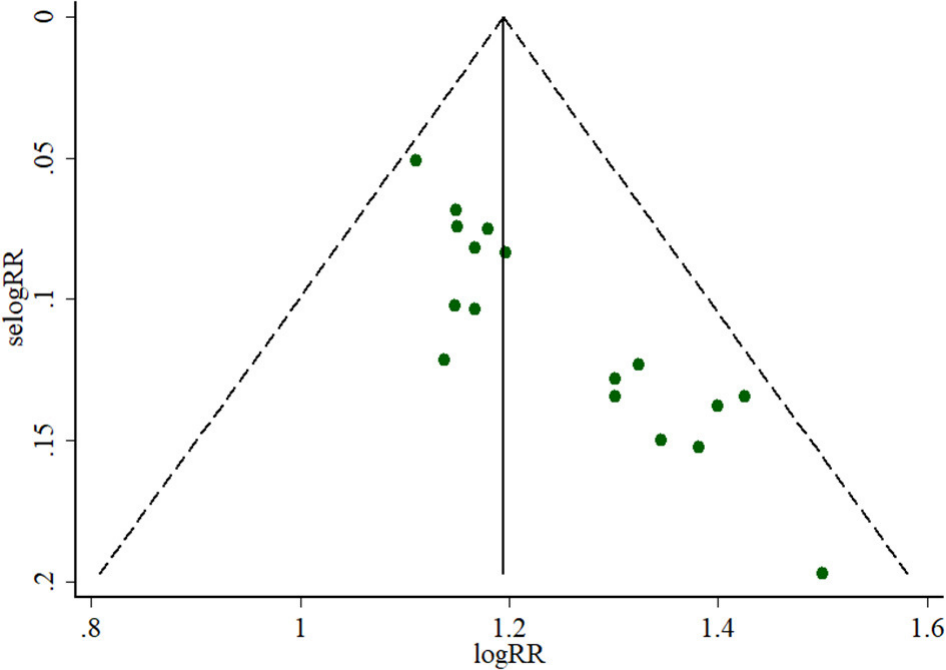
Funnel plot of outcome measure symptoms.

Angina symptoms were selected as the outcome measure in 17 RCTs. As shown in Figure 2, results from this meta-analysis showed that the pooled RR was 1.22 (95% CI 1.16 to 1.28; *Z* = 8.11, *p* < 0.00001) among the 17 RCTs, which suggested that using puerarin injection as adjunctive therapy was more effective than using Western medicine alone in treating UAP. Due to the low heterogeneity (*p* = 0.77, *I*^2^ = 0%), the fixed-effect model was employed in this meta-analysis. As shown in Figure 3, the potential publication bias among the 17 RCTs was assessed by funnel plots and results suggested that slight asymmetry was observed. Results from Begg's test (*Z* = 3.75, *p* = 0) and Egger's test (*t* = 7.25, *p* = 0) also indicated that there was publication bias. To evaluate whether the overall effect of puerarin injection as adjunctive therapy superior to Western medicine alone in treating UAP would be affected by the low-quality RCTs, a sensitivity analysis was conducted. As shown in Table 3, no obvious difference was observed when low-quality RCTs were gradually excluded according to the M scale. Slight changes (0.02 in magnitude) were observed between the high-quality (M scale > 3) and low-quality (M scale ≤ 3) RCTs. To evaluate whether the overall effect was affected by sample size, publication date, and dosages of puerarin injection among the included RCTs, subgroup analysis was conducted (Supplementary Table 2). No significant difference was observed in the pooled RRs, which consistently demonstrated that the therapeutic efficacy of puerarin injection as adjunctive therapy surpassed Western medicine alone in treating UAP.

**Table 3:** Sensitivity analysis based on the outcome measure symptoms.

	**Group**	**No. of RCTs**	**No. of patients**	**RR**	**95%CI**	**Z**	***P*** **(effect)**	**I^**2**^**	**χ^2^**	**P (het)**
M scale	0–5	17	1,459	1.22	1.16, 1.28	8.11	<0.00001	0%	11.55	0.77
	1–5	17	1,459	1.22	1.16, 1.28	8.11	<0.00001	0%	11.55	0.77
	2–5	17	1,459	1.22	1.16, 1.28	8.11	<0.00001	0%	11.55	0.77
	3–5	17	1,459	1.22	1.16, 1.28	8.11	<0.00001	0%	11.55	0.77
	4–5	7	559	1.23	1.14, 1.33	5.18	<0.00001	0%	3.26	0.78
	≤ 3	10	900	1.21	1.14, 1.29	6.25	<0.00001	0%	8.07	0.53
	> 3	7	559	1.23	1.14, 1.33	5.18	<0.00001	0%	3.26	0.78

### Electrocardiogram Improvement

**Figure 4:**
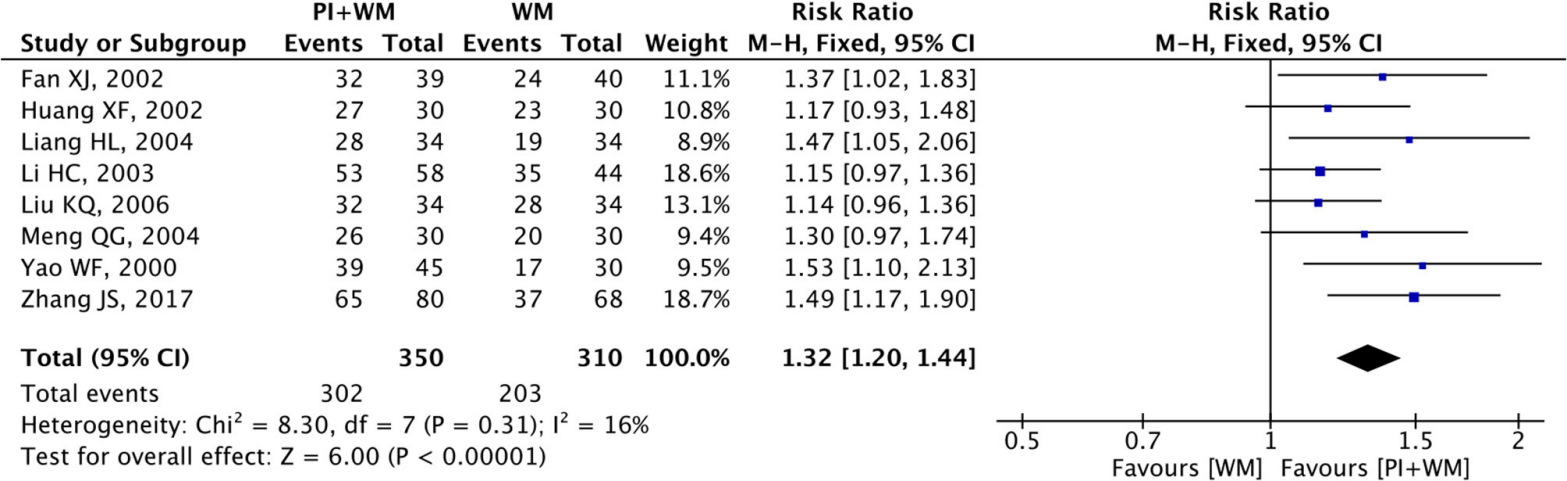
Forest plot of outcome measure ECG improvement. PI is puerarin injection and WM is Western medicine.

**Figure 5:**
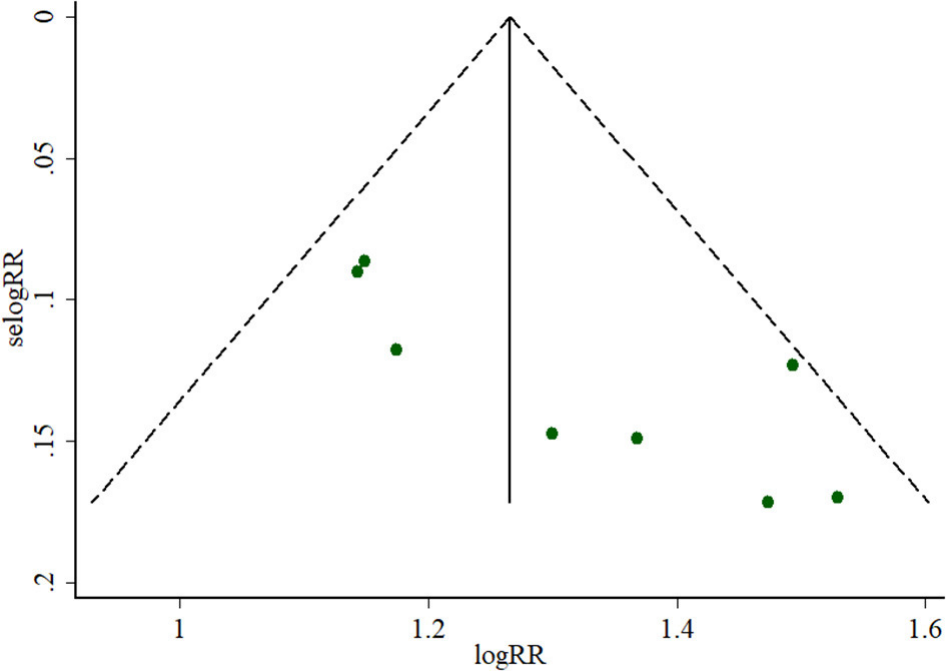
Funnel plot of outcome measure ECG.

Electrocardiogram improvement was selected as the outcome measure in eight RCTs. The fixed-effect model was employed to analyze the collected data since there was no significant heterogeneity (*p* = 0.31, *I*^2^ = 16%). As shown in Figure 4, the pooled RR was 1.32 (95% CI 1.20 to 1.44; *Z* = 6.00, *p* < 0.00001) among the eight RCTs, which indicated puerarin injection as adjunctive therapy was more effective than Western medicine alone in the improvement for ECG. To evaluate whether the overall effect of puerarin injection as adjunctive therapy was more effective than Western medicine alone in ECG improvement were affected by low-quality RCTs, sensitivity analysis was conducted in this meta-analysis. As shown in Table 4, no significant difference was observed when low-quality RCTs were gradually excluded according to the M scale. Slight changes (0.05 in magnitude) were observed for the included RCTs with high quality (M scale > 3) and low quality (M scale ≤ 3). To evaluate whether the overall effect was affected by sample size, publication date, and dosages of puerarin injection among the eight RCTs, subgroup analysis was conducted (Supplementary Table 3). No significant difference was observed in the pooled RRs, which consistently demonstrated that puerarin injection as adjunctive therapy was better than Western medicine alone in treating UAP. The potential publication bias among the eight RCTs was assessed by funnel plots, as shown in Figure 5, results suggested that slight asymmetry was observed. Results from Begg's test (*Z* = 2.10, *p* = 0.035) and Egger's test (*t* = 3.72, *p* = 0.010) also indicated that there was publication bias.

**Table 4:** Sensitivity analysis based on the outcome measure ECG.

	**Group**	**No. of RCTs**	**No. of patients**	**RR**	**95%CI**	**Z**	**P (effect)**	**I^**2**^**	**χ^2^**	**P (het)**
M scale	0–5	8	660	1.32	1.20, 1.44	6.00	<0.00001	16%	8.30	0.31
	1–5	8	660	1.32	1.20, 1.44	6.00	<0.00001	16%	8.30	0.31
	2–5	8	660	1.32	1.20, 1.44	6.00	<0.00001	16%	8.30	0.31
	3–5	8	660	1.32	1.20, 1.44	6.00	<0.00001	16%	8.30	0.31
	4–5	4	282	1.29	1.13, 1.47	3.83	0.0001	16%	3.58	0.31
	≤ 3	4	378	1.34	1.18, 1.52	4.62	<0.00001	31%	4.33	0.23
	> 3	4	282	1.29	1.13, 1.47	3.83	0.0001	16%	3.58	0.31

### Frequency of Angina Attack

**Figure 6:**
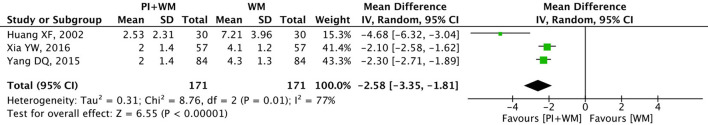
Funnel plot on the frequency of angina attack.

Three included RCTs reported the frequency of angina attacks (time/week). The random-effect model was used to analyze the data since there was significant heterogeneity (*I*^2^ = 77%, *p* = 0.01). As shown in Figure 6, results suggested that the adjunctive use of puerarin injection was better than Western medicine alone in reducing the frequency of angina attacks (MD = −2.58; 95% CI −3.35 to −1.81; *Z* = 6.55, *p* < 0.00001). To evaluate if the overall effect in reducing the frequency of angina attack was influenced by low-quality RCTs, sensitivity analysis was performed. As shown in Table 5, there was a significant difference when the low-quality RCTs were gradually excluded based on the M scale. Big changes (2.46 in magnitude) were found for the included RCTs with high quality (M scale > 3) and low quality (M scale ≤ 3). To assess if the overall effect was influenced by sample size and publication date among the three RCTs, subgroup analysis was introduced in this meta-analysis (Supplementary Table 4). A significant difference was observed in the overall MDs. After carefully reading the original RCT (23), it was found that the 26 patients with initial onset angina pectoris could be the important reason leading to heterogeneity. Hence, we removed this RCT and pooled other two RCTs alone (MD = −2.22, 95% CI −2.53 to −1.90, *Z* = 13.97, *I*^2^ = 0%, *p* < 0.00001).

**Table 5:** Sensitivity analysis based on the frequency of angina attacks.

	**Group**	**No. of RCTs**	**No. of patients**	**MD**	**95%CI**	**Z**	***p*** **(effect)**	**I^**2**^**	**χ^2^**	***p*** **(het)**
M scale	0–5	3	342	−2.58	−3.35, −1.81	6.55	<0.00001	77%	8.76	0.01
	1–5	3	342	−2.58	−3.35, −1.81	6.55	<0.00001	77%	8.76	0.01
	2–5	3	342	−2.58	−3.35, −1.81	6.55	<0.00001	77%	8.76	0.01
	3–5	3	342	−2.58	−3.35, −1.81	6.55	<0.00001	77%	8.76	0.01
	4–5	1	60	−4.68	−6.32, −3.04	5.59	<0.00001	/	/	/
	≤ 3	2	282	−2.22	−2.53, −1.90	13.97	<0.00001	0%	0.39	0.53
	> 3	1	60	−4.68	−6.32, −3.04	5.59	<0.00001	/	/	/

### Duration of Angina Attack

**Figure 7:**
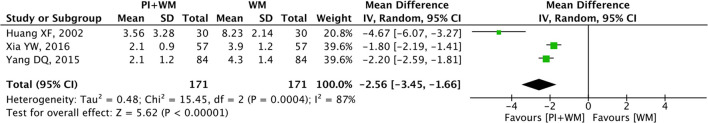
Funnel plot on the duration of angina attack.

Duration of angina attacks (minutes for each attack) was reported in three included RCTs in this meta-analysis. There was significant heterogeneity (*I*^2^ = 87%, *p* = 0.0004) and random effect model was adopted. As shown in Figure 7, results indicated that puerarin injection as adjunctive therapy had a better therapeutical effect in reducing the duration of angina attacks than Western medicine alone (MD = −2.56, 95% CI −3.45 to −1.66, *Z* = 5.62, *p* < 0.00001). To evaluate if the overall effect of MDs in reducing the duration of angina attack was influenced by low-quality RCTs, sensitivity analysis was performed. As shown in Table 6, a significant difference was observed when the low-quality RCTs were gradually excluded. Significant changes (2.67 in magnitude) were found for the included three RCTs with high quality (M scale > 3) and low quality (M scale ≤ 3). To assess if the overall effect of MDs was influenced by sample size and publication date among the three RCTs, subgroup analysis was introduced in this meta-analysis (Supplementary Table 5). A significant difference was observed in the overall MDs. For the same reason, the heterogeneity was attributed to the patients with initial onset angina pectoris in the RCT (23). Finally, this RCT was removed and other two RCTs were pooled alone (MD = −2.00, 95% CI −2.39 to −1.61, *Z* = 9.99, *I*^2^ = 50%, *p* < 0.00001).

**Table 6:** Sensitivity analysis based on the duration of angina attacks.

	**Group**	**No. of RCTs**	**No. of patients**	**MD**	**95%CI**	**Z**	***P*** **(effect)**	**I^**2**^**	**χ^2^**	***P*** **(het)**
M scale	0–5	3	342	−2.56	−3.45, −1.66	5.62	<0.00001	87%	15.45	0.0004
	1–5	3	342	−2.56	−3.45, −1.66	5.62	<0.00001	87%	15.45	0.0004
	2–5	3	342	−2.56	−3.45, −1.66	5.62	<0.00001	87%	15.45	0.0004
	3–5	3	342	−2.56	−3.45, −1.66	5.62	<0.00001	87%	15.45	0.0004
	4–5	1	60	−4.67	−6.07, −3.27	6.53	<0.00001	/	/	/
	≤ 3	4	282	−2.00	−2.39, −1.61	9.99	<0.00001	50%	2.00	0.16
	> 3	1	60	−4.67	−6.07, −3.27	6.53	<0.00001	/	/	/

### Adverse Reaction

Six included RCTs reported adverse reactions and one study described that no adverse reaction happened during the treatment. The rest of the studies did not report adverse reactions. As shown in Table 7, puerarin injection as adjunctive therapy could cause headache (3 cases), fever (5 cases), abdominal distension (4 cases), pruritus (3 cases), nausea (1 case), orthostatic hypotension (1 case), dizzy (1 case), and perspiration (1 case). No adverse reaction was reported in the control group (Western medicine alone). Although the adjunctive use of puerarin injection could cause adverse reactions for the UAP patients, none of the reported adverse reactions were serious. Hence, puerarin injection was safe in treating UAP.

**Table 7:** Incidence rate of adverse reaction.

**Type**	**Number of adverse reactions**		**References**
	**Treatment group**	**Control group**	
Headache	3	0	(19, 22)
Fever	5	0	(19, 21, 23)
Abdominal distension	4	0	(21)
Pruritus	3	0	(21, 31)
Nausea	1	0	(23)
Orthostatic hypotension	1	0	(31)
Dizzy	1	0	(23)
Perspiration	1	0	(25)

### GRADE Assessment

The GRADE criteria were employed to evaluate the evidence quality for the two outcomes (symptom and ECG). Since only two RCTs reported the duration and frequency of angina attack in this study, the GRADE assessment for these two outcomes did not provide. Results indicated low quality with serious methodological problems (risk of bias and reporting bias) was found among the two outcomes. The profile of GRADE evidence was shown in Table 8.

**Table 8:** GRADE evidence profile.

**Quality assessment**							**No of patients**		**RR/WMD (95% CI)**	***P* value**	**Quality**
**No of studies**	**Design**	**Risk of bias**	**Inconsistency**	**Indirectness**	**Imprecision**	**Other considerations**	**PI+WM**	**WM**			
**Symptom**											
17	RCT	serious^a, b, c^	No serious inconsistency	No serious indirectness	No serious imprecision	Reporting bias^d^	678/743 (91.3%)	537/716 (75%)	RR = 1.22 (1.16–1.28)	<0.00001	⊕⊕°° LOW
								72.5%			
**ECG**											
8	RCT	serious^a, b, c^	No serious inconsistency	No serious indirectness	No serious imprecision	Reporting bias^d^	302/350 (86.3%)	203/310 (65.5%)	RR = 1.32 (1.2–1.44)	<0.00001	⊕⊕°° LOW
								63.3%			

*PI, Puerarin injection; WM, Western medicine; CI, Confidence interval; RR, relative risks; WMD, weight mean difference;*

a *No details of random protocol were reported;*

b *lack of allocation concealment;*

c *Didn't report the implementation of blinding;*

d *Quantitative evaluation of the included data indicated publication bias*.

## Discussion

As a serious clinical syndrome subset of the acute coronary syndromes, UAP can easily deteriorate into sudden death (3). Conventional Western medicines, namely, calcium antagonists, β-blockers, and nitrates have been successfully employed to decrease anginal attacks. However, these medicines might have unsatisfied therapeutic effects for UAP due to their potential adverse reactions and drug resistances (4, 5). Puerarin injection as adjunctive therapy could be an effective way to provide better therapeutic effects for UAP since it can protect against myocardial ischemia/reperfusion injury by regulating the inflammatory response and NLRP3 inflammasome, which could be a good candidate to treat ischemic heart disease (36). So far, a large number of studies reported that the combined use of puerarin injection and conventional Western medicines presented positive therapeutic effects in treating UAP. However, the efficacy of puerarin injection for UAP has not been well verified at present. Recently, a meta-analysis and systematic review were performed for evaluating the clinical efficacy and safety of puerarin injection on the treatment of UAP (37). Although results from their study showed that puerarin injection in combination with conventional Western medicines exhibited significant improvements in the incidence of angina pectoris, electrocardiogram findings, nitroglycerin consumption, and plasma endothelin levels, several limitations still existed in their research. First, in this study, the meta-analysis and systematic review were not strictly designed according to the PRISMA statements. Second, the formulation and dosages of Western medicines in the control group were not described. Third, the detailed results of sensitivity analysis were lacking. Besides, subgroup analysis had not been conducted in their study. Fourth, only funnel plots were performed to evaluate the potential publication bias without Begg's test and Egger's test, which might cause inaccurate results. Hence, this study aimed to conduct a comprehensive and the PRISMA-compliant systematic review with sensitivity and subgroup analysis to evaluate the efficacy of puerarin injection as adjunctive therapy in treating UAP, providing a more reliable conclusion for puerarin injection.

### Analysis of Effectiveness

After analysis of the RCTs on puerarin injection, the therapeutic efficacy of puerarin injection in treating UAP was proved to be effective. First, the combination use of UAP and Western medicines could improve the total effective rate of angina symptoms and ECG. In this meta-analysis, puerarin injection as adjunctive therapy displayed better therapeutic effects than Western medicine alone in reducing angina symptoms (RR 1.22, 95% CI 1.16 to 1.28, *Z* = 8.11, *p* < 0.00001) and improving ECG (RR 1.32, 95% CI 1.20 to 1.44, *Z* = 6.00, *p* < 0.00001), which indicated that puerarin injection as adjunctive therapy could provide better efficacy than Western medicine alone in treating UAP. This results met with the trends of the previous study (reduction in the number of angina pectoris attacks (RR 1.29, 95% CI 1.24 to −1.34, *Z* = 13.07, *p* < 0.001) and ECG improvement (RR 1.33, 95% CI 1.27 to 1.39, *Z* = 12.30, *p* < 0.001) (37). Second, this study demonstrated that the adjunctive therapy of puerarin injection for UAP could reduce the frequency and duration of angina attack, which had not been evaluated in the previous study (37). Third, in the study of Gao et al. (37), they found that the major adverse reactions included transient headache and fever, dizziness, hypotension, abdominal distension and nausea, allergic skin reactions, and sinus bradycardia. Moreover, all of the adverse reactions were mild, with spontaneous remission. In this study, several adverse reactions, namely, headache, fever, abdominal distension, pruritus, nausea, orthostatic hypotension, dizziness, and perspiration were observed. However, none of these adverse reactions were serious, which also consistently suggested that puerarin injection as adjunctive therapy was safe for the treatment of UAP. The good therapeutic efficacy of puerarin injection on UAP could be contributed to the numerous effects on the cardiovascular system. Previous studies have demonstrated that puerarin injection was beneficial for the cardiovascular system by expanding the coronary artery to relieve vasospasm and increasing coronary blood flow, thus improving the blood supply to the ischemic myocardium (8). Besides, it can decrease blood pressure, heart rate, and myocardial oxygen consumption (9). Puerarin injection also plays important role in inhibiting platelet aggregation, decreasing blood viscosity, and improving microcirculation (10). The pharmacological effects of puerarin on the cardiovascular system have been summarized as follows: (1) effects of antiarrhythmia: antiarrhythmia effects of puerarin could be partly attributed to its influences on Na^+^ and K^+^ channels. Previous studies have demonstrated that puerarin could inhibit K^+^ channels, which could prolong the duration of the action potential and the refractory period in cardiomyocytes to prevent arrhythmia development (38). Besides, puerarin could block Na^+^ channels, which could significantly suppress the velocity of cardiac impulse conduction and prolong the refractory period of cardiac excitability, thus inhibiting the progression of arrhythmia (39). (2) Improving microcirculation: puerarin could improve myocardial microcirculation and cardiac performance by downregulating the endothelin system and normalizing the expression of SERCA2a and phospholamban (40). (3) Protection of ischemia-reperfusion: it had been demonstrated that puerarin had protective functions on transient spinal cord ischemia-reperfusion injury in rabbits and rats by increasing the transcription of thioredoxin and reducing apoptosis (41). (4) Decreasing heart rate: puerarin could reduce systolic blood pressure and heart rate by blocking the P2X3 signaling transmission and then depress the aggravated sympathoexcitatory reflex to relieve myocardial ischemic damage (42). (5) Inhibiting cardiac hypertrophy: puerarin could suppress angiotensin II-induced cardiac hypertrophy by blocking the excessive generation of reactive oxygen species (ROS) and disrupting the downstream activation of the p38, ERK1/2, and nuclear factor-kappa B (NF-κB) pathways (43).

### Strengths and Limitations

A large number of studies have reported that puerarin injection could improve the therapeutic efficacy of Western medicine in the treatment of UAP. However, the efficacy of puerarin injection as adjunctive therapy for UAP had not been verified. This study aimed to provide the PRISMA-compliant systematic review and meta-analysis to assess the efficacy of puerarin injection as adjunctive therapy in treating UAP (Supplementary Table 6). Although the benefits of puerarin injection for UAP have been proved by this meta-analysis, several limitations still existed. First, the methodological quality of several RCTs is low (only three RCTs scored 3 scores with the Jadad scale). Randomization is considered to be an effective way to avoid selection bias, while only three RCTs described the appropriate randomization method. Moreover, blinding for researchers or patients was not mentioned in all RCTs. These shortcomings could cause an overestimation or underestimation result for the adjunctive therapy in treating UAP. Second, the sample size of some RCTs was relatively small and the duration of treatment was relatively short, which might cause an inaccurate result for this meta-analysis. Therefore, more strictly designed RCTs with higher quality, larger sample size, and longer duration are urgent to be recommended to evaluate the efficacy of puerarin injection as adjunctive therapy for UAP. Third, the result from the GRADE system for the outcomes symptom and ECG indicated that the quality of evidence was low. More high-quality RCTs were recommended to verify the efficacy of puerarin injection as adjunctive therapy for UAP.

### Implications for Further Research

Although the efficacy of puerarin injection as adjunctive therapy for UAP has been verified in this meta-analysis, the evidence was still insufficient due to the low-quality RCTs. Hence, several suggestions were recommended to conduct the studies on puerarin injection. First, studies on puerarin injection should be strictly designed according to the Consolidated Standards of Reporting Trials (CONSORT) statement. Second, to provide a reliable result for puerarin injection, more RCTs with multicenter, higher methodological quality, larger sample size, and longer treatment duration were still demanded.

## Conclusion

This meta-analysis suggested that puerarin injection as adjunctive therapy was superior to Western medicine alone in treating UAP. However, due to the low-quality RCTs, more strictly designed RCTs with higher methodological quality, larger sample size, and longer treatment duration are still recommended to provide reliable evidence for puerarin injection.

## Data Availability Statement

The original contributions presented in the study are included in the article/Supplementary Material, further inquiries can be directed to the corresponding author/s.

## Author Contributions

HS: design, conception, searching the literature, extracting data, evaluating quality, performing statistical analysis, interpreting data, and drafting and revising the manuscript. YH: searching the literature, extracting data, evaluating quality, performing statistical analysis, interpreting data, and drafting the manuscript. DX: searching the literature, extracting data, evaluating quality, interpreting data, and drafting the manuscript. SH: design, conception, performing statistical analysis, interpreting data, and drafting and revising the manuscript. RT: design, conception, and revising manuscript. All authors approved the final version of the manuscript.

## Funding

This work was supported by the National Key Research and Development Program of China (2020YFC2005500), the Key Research and Development Program of Science and Technology Department of Sichuan Province (2019YFS0514), the China Postdoctoral Science Foundation (2021M690559), and the Young Talents Project of Sichuan Academy of Medical Sciences and Sichuan Provincial People's Hospital (Grant: 2021QN07).

## Conflict of Interest

The authors declare that the research was conducted in the absence of any commercial or financial relationships that could be construed as a potential conflict of interest.

## Publisher's Note

All claims expressed in this article are solely those of the authors and do not necessarily represent those of their affiliated organizations, or those of the publisher, the editors and the reviewers. Any product that may be evaluated in this article, or claim that may be made by its manufacturer, is not guaranteed or endorsed by the publisher.
