# Supplementary material for: A Systematic Review and Meta-Analysis on the Efficacy of Puerarin Injection as Adjunctive Therapy for Unstable Angina Pectoris
Source: Front Cardiovasc Med. 2022 Feb 24;9:763567. doi: 10.3389/fcvm.2022.763567 (PMC8907531; doi:10.3389/fcvm.2022.763567)
Supplement: Supplementary file 1 [file Data_Sheet_1.docx]

**Supporting information:**

**
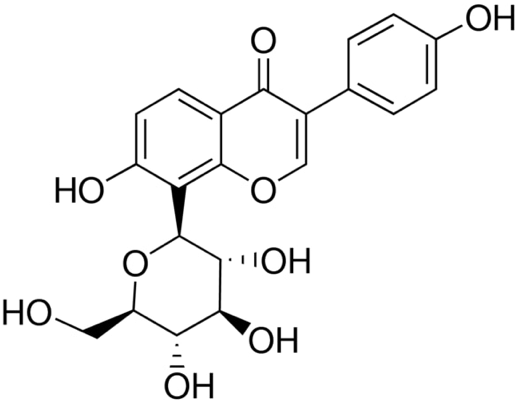
**

**Fig. S1** Structure of puerarin.

**Table S1.** Jadad scale checklist

| Items |  | Score Standard |  |
| --- | --- | --- | --- |
|  | 0 | 1 | 2 |
| Randomization | Not randomized or inappropriate method of randomization. | The study was as randomized. | The method of randomized was described and it was appropriate. |
| Double blinding | No blind or inappropriate method of double blinding. | The study was as double blind. | The method of double blinding was described and it was appropriate. |
| Withdrawals and dropouts | Not describe the follow-up. | A description of withdrawals and dropout. |  |

**Table S2.** Subgroup analysis based on the outcome measure symptoms.

|  | Group | No. of RCTs | No. of patients | RR | 95%CI | Z | P(effect) | I^2^ | ﻿χ^2^ | P(het) |
| --- | --- | --- | --- | --- | --- | --- | --- | --- | --- | --- |
| ﻿Sample size | < 70 | 7 | 416 | 1.30 | 1.18, 1.44 | 5.21 | < 0.00001 | 0% | 3.63 | 0.73 |
|  | ≥ 70 | 10 | 1043 | 1.19 | 1.13, 1.25 | 6.25 | < 0.00001 | 0% | 5.18 | 0.82 |
| Year of publication | < 2006 | 9 | 618 | 1.31 | 1.20, 1.42 | 6.32 | < 0.00001 | 0% | 4.41 | 0.82 |
|  | ≥ 2006 | 8 | 841 | 1.16 | 1.10, 1.23 | 5.14 | < 0.00001 | 0% | 2.09 | 0.95 |
| Dosage of  puerarin | < 500 mg | 8 | 627 | 1.27 | 1.18, 1.37 | 6.25 | < 0.00001 | 0% | 5.08 | 0.65 |
|  | ≥ 500 mg | 9 | 832 | 1.18 | 1.11, 1.26 | 5.29 | < 0.00001 | 0% | 4.39 | 0.82 |

**Table S3.** Subgroup analysis based on the outcome measure ECG.

|  | Group | No. of RCTs | No. of patients | RR | 95%CI | Z | P(effect) | I^2^ | ﻿χ^2^ | P(het) |
| --- | --- | --- | --- | --- | --- | --- | --- | --- | --- | --- |
| ﻿Sample size | < 70 | 4 | 256 | 1.26 | 1.11, 1.43 | 3.50 | 0.0005 | 0% | 2.34 | 0.51 |
|  | ≥ 70 | 4 | 404 | 1.36 | 1.20, 1.55 | 4.87 | < 0.00001 | 39% | 4.95 | 0.18 |
| Year of publication | < 2006 | 6 | 444 | 1.30 | 1.17, 1.45 | 4.81 | < 0.00001 | 0% | 4.45 | 0.49 |
|  | ≥ 2006 | 2 | 216 | 1.29 | 0.96, 1.75 | 1.68 | 0.09 | 75% | 4.06 | 0.04 |
| Dosage of  puerarin | < 500 mg | 3 | 230 | 1.27 | 1.07, 1.46 | 3.27 | 0.001 | 3% | 2.07 | 0.35 |
|  | ≥ 500 mg | 5 | 430 | 1.35 | 1.20, 1.52 | 5.02 | < 0.00001 | 34% | 6.02 | 0.20 |

**Table S4.** Subgroup analysis based on the frequency of angina attacks.

|  | Group | No. of RCTs | No. of patients | MD | 95%CI | Z | P(effect) | I^2^ | ﻿χ^2^ | P(het) |
| --- | --- | --- | --- | --- | --- | --- | --- | --- | --- | --- |
| ﻿Sample size | < 70 | 1 | 60 | -4.68 | -6.32, -3.04 | 5.59 | < 0.00001 | / | / | / |
|  | ≥ 70 | 2 | 282 | -2.22 | -2.53, -1.90 | 13.97 | < 0.00001 | 0% | 0.39 | 0.53 |
| Year of publication | < 2006 | 1 | 60 | -4.68 | -6.32, -3.04 | 5.59 | < 0.00001 | / | / | / |
|  | ≥ 2006 | 2 | 282 | -2.22 | -2.53, -1.90 | 13.97 | < 0.00001 | 0% | 0.39 | 0.53 |

**Table S5.** Subgroup analysis based on the duration of angina attacks.

|  | Group | No. of RCTs | No. of patients | MD | 95%CI | Z | P(effect) | I^2^ | ﻿χ^2^ | P(het) |
| --- | --- | --- | --- | --- | --- | --- | --- | --- | --- | --- |
| ﻿Sample size | < 70 | 1 | 60 | -4.67 | -6.07, -3.27 | 6.53 | < 0.00001 | / | / | / |
|  | ≥ 70 | 2 | 282 | -2.00 | -2.39, -1.61 | 9.99 | < 0.00001 | 50% | 2.00 | 0.16 |
| Year of publication | < 2006 | 1 | 60 | -4.67 | -6.07, -3.27 | 6.53 | < 0.00001 | / | / | / |
|  | ≥ 2006 | 2 | 282 | -2.00 | -2.39, -1.61 | 9.99 | < 0.00001 | 50% | 2.00 | 0.16 |

**Table S6.** Checklists of items to include when reporting a systematic review or meta-analysis.

| **Section/topic** | **#** | | | **Checklist item** | **Section &/or figure reported in:** |
| --- | --- | --- | --- | --- | --- |
| **TITLE** | | | | |  |
| Title | 1 | | | Identify the report as a systematic review, meta-analysis, or both. | Title |
| **ABSTRACT** | | | | |  |
| Structured summary | 2 | | | Provide a structured summary including, as applicable: background; objectives; data sources; study eligibility criteria, participants, and interventions; study appraisal and synthesis methods; results; limitations; conclusions and implications of key findings; systematic review registration number. | Abstract |
| **INTRODUCTION** | | | | |  |
| Rationale | 3 | | | Describe the rationale for the review in the context of what is already known. | Introduction |
| Objectives | 4 | | | Provide an explicit statement of questions being addressed with reference to participants, interventions, comparisons, outcomes, and study design (PICOS). | Introduction |
| **METHODS** | | | | |  |
| Protocol and registration | 5 | | | Indicate if a review protocol exists, if and where it can be accessed (e.g., Web address), and, if available, provide registration information including registration number. | No |
| Eligibility criteria | 6 | | | Specify study characteristics (e.g., PICOS, length of follow-up) and report characteristics (e.g., years considered, language, publication status) used as criteria for eligibility, giving rationale. | Methods-Eligibility criteria |
| Information sources | 7 | | | Describe all information sources (e.g., databases with dates of coverage, contact with study authors to identify additional studies) in the search and date last searched. | Methods-Information sources |
| Search | 8 | | | Present full electronic search strategy for at least one database, including any limits used, such that it could be repeated. | Methods-Search strategies |
| Study selection | 9 | | | State the process for selecting studies (i.e., screening, eligibility, included in systematic review, and, if applicable, included in the meta-analysis). | Methods-Study selection |
| Data collection process | 10 | | | Describe method of data extraction from reports (e.g., piloted forms, independently, in duplicate) and any processes for obtaining and confirming data from investigators. | Methods-Data collection process |
| Data items | 11 | | | List and define all variables for which data were sought (e.g., PICOS, funding sources) and any assumptions and simplifications made. | Methods-Data items |
| Risk of bias in individual studies | 12 | | | Describe methods used for assessing risk of bias of individual studies (including specification of whether this was done at the study or outcome level), and how this information is to be used in any data synthesis. | Methods-Quality assessment |
| Summary measures | 13 | | | State the principal summary measures (e.g., risk ratio, difference in means). | Methods-Statistical analysis |
| Synthesis of results | 14 | | | Describe the methods of handling data and combining results of studies, if done, including measures of consistency (e.g., I^2^) for each meta-analysis. | Methods-Statistical analysis |
| Risk of bias across studies | 15 | | | Specify any assessment of risk of bias that may affect the cumulative evidence (e.g., publication bias, selective reporting within studies). | Methods-Risk of bias across studies |
| Additional analyses | 16 | | | Describe methods of additional analyses (e.g., sensitivity or subgroup analyses, meta-regression), if done, indicating which were pre-specified. | Methods-Sensitivity and subgroup analysis |
| **RESULTS** | | | | | |
| Study selection | | 17 | | Give numbers of studies screened, assessed for eligibility, and included in the review, with reasons for exclusions at each stage, ideally with a flow diagram. | Results-Study selection |
| Study characteristics | | 18 | | For each study, present characteristics for which data were extracted (e.g., study size, PICOS, follow-up period) and provide the citations. | Results-Study characteristics |
| Risk of bias within studies | | 19 | | Present data on risk of bias of each study and, if available, any outcome level assessment (see item 12). | Results-Risk of bias in individual studies |
| Results of individual studies | | 20 | | For all outcomes considered (benefits or harms), present, for each study: (a) simple summary data for each intervention group (b) effect estimates and confidence intervals, ideally with a forest plot. | Result-Results of individual studies and their synthesis |
| Synthesis of results | | 21 | | Present results of each meta-analysis done, including confidence intervals and measures of consistency. | Result-Results of individual studies and their synthesis |
| Risk of bias across studies | | 22 | | Present results of any assessment of risk of bias across studies (see Item 15). | Result-Risk of bias across studies |
| Additional analysis | | 23 | | Give results of additional analyses, if done (e.g., sensitivity or subgroup analyses, meta-regression [see Item 16]). | Result-Sensitivity and subgroup analysis |
| **DISCUSSION** | | | | | |
| Summary of evidence | | 24 | | Summarize the main findings including the strength of evidence for each main outcome; consider their relevance to key groups (e.g., healthcare providers, users, and policy makers). | Analysis of Effectiveness |
| Limitations | | 25 | | Discuss limitations at study and outcome level (e.g., risk of bias), and at review-level (e.g., incomplete retrieval of identified research, reporting bias). | Strength and Limitations |
| Conclusions | | 26 | | Provide a general interpretation of the results in the context of other evidence, and implications for future research. | Conclusions |
| **FUNDING** | | | | | |
| Funding | | | 27 | Describe sources of funding for the systematic review and other support (e.g., supply of data); role of funders for the systematic review. | Acknowledgments |
